# Welfare impacts of smallholder farmers’ participation in multiple output markets: Empirical evidence from Tanzania

**DOI:** 10.1371/journal.pone.0250848

**Published:** 2021-05-06

**Authors:** Julius Manda, Carlo Azzarri, Shiferaw Feleke, Bekele Kotu, Lieven Claessens, Mateete Bekunda

**Affiliations:** 1 International Institute of Tropical Agriculture (IITA), C/o World Vegetable Centre–Eastern and Southern Africa, Arusha, Tanzania; 2 International Food Policy Research Institute (IFPRI), Washington, DC, United States of America; 3 International Institute of Tropical Agriculture (IITA), Dares Salaam, Tanzania; 4 International Institute of Tropical Agriculture, Tamale, Ghana; Newcastle University, School of Natural and Environmental Sciences, UNITED KINGDOM

## Abstract

A relatively large body of literature has documented the welfare effects of smallholder farmers’ participation in single-commodity output markets. However, limited empirical evidence is available when smallholder farmers participate in multiple-commodities output markets. We tried to fill this gap in the literature by estimating the impacts of smallholder farmers’ contemporaneous participation in both maize and legume markets *vis-à-vis* in only maize or legume markets using household-level data from Tanzania. Applying a multinomial endogenous switching regression model that allows controlling for observed and unobserved heterogeneity associated with market participation in single-commodity and multiple-commodity markets, results showed that smallholder farmers’ participation in both single–and multiple–commodity markets was positively and significantly associated with household income and food security. Moreover, the greatest benefits were obtained when farmers participated in multiple-commodity markets, suggesting the importance of policies promoting diversification in crop income sources to increase welfare and food security. Our findings also signal the complementary–rather than substitute–nature of accessing multiple-commodity markets for enhancing household livelihoods under a specialization strategy. Finally, important policy implications are suggested, from promoting and supporting public infrastructure investments to expanding road networks to reduce transportation costs, especially in remote communities, to enhance smallholder farmer access to profitable maize and legume markets in Tanzania.

## 1. Introduction

In Africa, south of the Sahara (SSA), North Africa, and the Middle East, more than 30% of the population shows poor market access and, specifically, SSA is immensely disadvantaged in infrastructure, thereby facing high transaction costs and market risks [1]. Good road infrastructure is often associated with better access to markets, which translates into lower transport costs, enhanced agricultural production, non-farm diversification, income, and food security [2–4]. The reduced transaction costs associated with better access to markets will inevitably lead to an increase in market participation, the number of crops produced, and the quantity of produce sold. Several studies support the positive relationship between market participation and household income [e.g., 5–9]. For example, [10] demonstrated that vegetable commercialization is positively and statistically significantly related to household income in Kenya. Similarly, [11] demonstrated that commercialization led to a reduction of income based as well as multidimensional poverty among smallholder farmers in Kenya. [6] also found that participation in the maize and pigeon pea markets in Tanzania had led to an increase in consumption expenditure, ranging from 19% to 29%.

While there are several studies on the household income effects of smallholder farmers’ participation, there are relatively few studies that have examined the relationship between market participation and food/nutrition security [e.g., 7–9, 12]. More importantly, most of these studies assessed the welfare impacts of smallholder farmers’ participation in a single-output market, overlooking the fact that most African smallholder farmers manage a farming system of multiple enterprises through interdependent decision-making process.

Maize and legumes are the most important staple commodities in Tanzania, with, maize accounting for nearly 33% of caloric intake. Tanzania is also the largest producer and net exporter of common beans in Africa [13, 14]. The synergy between the production of maize and legumes is not limited only to cash flow but also to soil nutrient flow as maize is intercropped or relay-cropped with legumes.

We specifically consider maize and legume markets as multiple-output markets because of the double role of these crops for home consumption and market sales. Smallholder farmers produce maize and legumes as a strategy of stable cash flow and risk management, regardless of their level of market integration. In the past, maize and legumes were produced primarily for home consumption and income sources, respectively. However, as smallholder farmers started using improved maize technologies and marketing production surplus, maize sales turned out to be a major income source, complementing income from legumes. For example, as legume harvest occurs before maize harvest, farmers can earn from legume sales so that they can decide to delay maize sales along the agricultural season, benefiting from higher maize prices later in the season given their relatively greater variability than legume prices. This strategy provides not only steadier cash flow, but also more stable maize availability for home consumption.

In this paper, we aim to fill this gap in the literature by assessing the impact of smallholder farmers’ participation in multiple-output markets compared to single-output markets using multiple outcome variables (total household expenditure, food expenditure, household dietary diversity [HDD], duration of food insecurity [months], and household food insecurity access scale [HFIAS]). We contribute to the literature by assessing the income and food security impacts of smallholder farmers’ contemporaneous participation in maize and legume markets *vis-à-vis* in only maize or legume market. To this end, we specify the instrumental variable (IV) based multinomial endogenous switching regression (MESR) model that allows controlling for observed and unobserved heterogeneity associated with market participation in single-output (maize or legume) and multiple-output (maize and legume) markets. The model is applied in a simultaneous framework using household-level data from Tanzania. This is a point of departure from most previous studies -e.g., [5, 6]-, which assessed the determinants and impacts of maize and pigeon pea market participation on consumption expenditure in Tanzania. As a robustness check for the MESR model, we also estimate the multivalued inverse probability weighted regression adjustment (MIPWRA) model. It provides efficient estimates by allowing the modelling of the outcome and the treatment equations while requiring that only one of the two models be correctly specified to consistently estimate the impact owing to their double-robust property [15].

The rest of the article is organized as follows. The next section describes the data and sampling strategy, while Section 3 presents the definitions of market participation, household income, and food security. Section 4 describes the conceptual and empirical frameworks. The penultimate section presents the results and discussion, and the last section draws conclusions and policy recommendations.

## 2. Data and sampling strategy

We use micro-level data from a sample of 810 farm households conducted in two districts (Babati and Kiteto) in Manyara region and one district (Kongwa) in Dodoma region of Tanzania in 2014, which is the baseline evaluation survey of the Africa Research In Sustainable Intensification for the Next Generation (Africa RISING) project. The survey, based on a cluster quasi-randomized control trial design, collected baseline information among three farmers’ groups with their associated household members: a) Africa RISING participant farmers -that is, farmers who directly participate in Africa RISING activities in different ways such as by hosting and/or managing on-farm trials-, including 435 households in seven intervention villages; b) Africa RISING non-participant farmers including 105 households in the same seven intervention villages; and c) control farmers including 270 households in 18 non-intervention villages. Non-intervention villages were selected following a constrained randomization, hence randomly chosen among the universe of villages within the same agro-ecological zone as the seven intervention villages, but far from them to prevent the possibility of contamination. The household questionnaire was a multi-topic instrument specifically designed to collect information on the project’s core topics, such as food security and nutrition, poverty, livelihoods, agricultural production, productivity, and practices. The survey instrument was administered in two visits using computer-assisted personal interviewing (CAPI).

### 2.1 Ethics statement

“Data were collected using a household survey and were analyzed anonymously. Survey participants were randomly selected among the Africa RISING project beneficiaries and control group. All participants received a clear explanation of the survey objectives and were asked for their verbal informed consent to willingly participate in the study. If respondents declined to be interviewed, the reasons for their refusal were also recorded, and no respondent was forced to participate in the survey. Prior to conducting the study, the International Food Policy Research Institute (IFPRI) Internal Review Board approved the study on 8/21/2013 with the approval letter available upon request."

## 3. Measuring market participation, income, and food security

### 3.1 Market participation

In line with the theoretical market participation model developed by [16], a household is classified as a market participant if any of its members has sold any positive amount of maize and legumes during the last cropping season. Participation in maize and legume markets results in four (2^2^) different market choices i.e. non-market participation (M_0_L_0_), participation in maize market only (M_1_L_0_), participation in legume market only (M_0_L_1_), and participation in both maize and legume markets (M_1_L_1_) (Table 1). Legumes include groundnut, common beans and all the remaining pulses (e.g., soybean, pigeon pea, chickpeas etc.).

**Table 1:** Market participation choices.

Participation choice set	Combination	Maize market participation (M)		Legume market participation (L)		Frequency	Per cent
		M_0_	M_1_	L_0_	L_1_		
1	M_0_L_0_	√		√		230	28.40
2	M_1_L_0_		√	√		161	19.88
3	M_0_L_1_	√			√	91	11.23
4	M_1_L_1_		√		√	328	40.49

Note: M_0_L_0_: non-market participation; M_1_L_0_: only maize market participation; M_0_L_1_: only legume market participation; M_1_L_1_: joint maize and legume markets participation.

On average, about 28% of the households participated neither in maize nor in legume market, while 40% participated in both maize and legume markets. Relatively few farmers sold legumes (11%) compared with 20% of the farmers who sold maize, and this finding may also have implications for income and food security.

### 3.2 Income and food security

In this study, we use total household expenditure as a proxy for household income. Total household expenditure includes food and non-food consumption expenditure incurred by the household during the previous 12 months. Household income is mainly used as an indicator of household wellbeing [e.g., 17–19], although some studies have used it as a food security indicator [e.g., 20, 21].

The Food and Agriculture Organization (FAO) defined food security as a **“**situation that exists when all people, at all times, have physical, social and economic access to sufficient, safe and nutritious food that meets their dietary needs and food preferences for an active and healthy life” [22]. This definition encompasses the four dimensions of food security, i.e., food availability, access, utilization, and stability. In our study, food security is measured by four indicators: household food expenditure, dietary diversity, number of months of food insecurity, and HFIAS. Food expenditure is an indicator of economic vulnerability: households that spend a large percentage of their income on food are more susceptible to food scarcity because a reduction in their income would most likely lead to a reduction in food consumption or quality of food eaten [23]. Food expenditure includes food purchased, own-consumption, and food received as gift or in-kind payment or exchange. Previous studies have used food expenditure as an indicator of food security [e.g., 24–26].

Dietary diversity is defined as the number of different unique food items or food groups consumed over a given reference period [27]. Dietary diversity was initially developed as an indicator of quantity and quality of food access [28], although it is also a proxy for diet quality [25, 29]. Some studies have used it even to measure food utilization [25]. In this study, we used the household dietary diversity score (HDDS) as an indicator of dietary diversity. As part of the survey, households were asked to report the food items they had consumed over the seven days before the interview. Items included cereals, roots and tubers, vegetables, livestock products, fruits, beverages, and condiments, classified into 12 food groups based on the guidelines provided by [29]. The HDDS expresses how many of the 12 food groups encompass food items consumed by any household member over the reference period. Hence, the HDDS ranges from 1 to 12.

The number of months of food insecurity measures the length of time during which the household had a shortage of food to feed its members [30]. This is considered a self-reported measure of food (in)security, is based on perceptions of a general condition rather than on quantitative measurement.

Finally, the HFIAS, developed through the Food and Nutrition Technical Assistance Project (FANTA), is one of the widely used measures of household access to food and the degree of anxiety involved in its acquisition [31]. The HFIAS questions capture information on food shortage, food quantity, and quality of diet to determine the status of household access to food, proxying the general experience of food insecurity in the household [32]. The HFIAS ranges from 0 to 27, such that the higher the score, the more severe the food insecurity experienced [31].

## 4. Conceptual and econometric framework

### 4.1 Conceptual framework

In many African countries, including Tanzania, smallholder farmers usually face imperfect input and output markets. Markets fail because farmers face proportional and fixed transaction costs such as long distances to the market, poor infrastructure that increase transportation costs, high marketing margins due to traders with local monopoly power, high search and recruitment costs and imperfect information, among others [33–35]. The differences in the marketing margins among smallholders arising from differential access to assets and services might explain the underlying heterogeneous market participation among them [36]. Because the transaction costs drive a wedge between household buying and selling prices [37], many households fail to participate in profitable markets. When households do not participate in markets, production and consumption decisions are non-separable [33].

Smallholders’ production and consumption decisions are non-separable because they produce both for consumption and sale, i.e., goods are both supplied and demanded by the same household. Thus, smallholders’ market participation decisions are best analyzed using non-separable household models. In a non-separable household model (as opposed to a separable model), the consumption and production decisions are linked through endogenous market prices and factors influencing transaction costs in the markets [35]. We, therefore, follow earlier work in the vein by [16, 33, 38] in viewing the decision to sell maize and legumes from the perspective of the non-separable household model, in which family members organize their labour to maximize utility over a bundle of consumption goods produced on the farm or purchased from the market, subject to an income constraint generated by a combination of farm production, sales, and non-farm earnings. According to [16], the decision to participate in the market may depend on public goods and services (e.g., a radio broadcast of prices that affects search costs and road accessibility to market), household characteristics (e.g., age, education, and sex), household assets and access to non-farm income inter alia.

Nevertheless, the decision to participate in maize and legume markets may be endogenous as farmers may self-select into market participation based on both observable and unobservable characteristics. These characteristics may be systematically correlated with the outcome variables of interest, thereby leading to biased estimates. To account for any possible endogeneity, we model the single and joint decisions to participate in maize and legume markets in a multinomial framework. Using the MESR model, we proceed in two steps. In the first step, the decision to participate in the market (single and joint) is modelled using a multinomial logit selection model. In the second step, the impacts on our outcome variables of interest are estimated using ordinary least squares (OLS) with selectivity correction terms.

It is envisaged that the cash income obtained from participating in maize and legume markets will contribute to household income, which will translate into more food purchases (quantity and diversity), thereby leading to improved household food security and nutrition [9, 39]. It is generally believed that market participation leads to specialization in producing crops (usually cash crops) where they have a comparative advantage. However, [40] showed that on-farm diversification through intercropping a food and cash crop reduced market transaction costs borne by rural households and communities. Similarly, [41] found that integration into output markets was positively associated with a diversification of land use away from rice monoculture in Thailand. Therefore, considering the numerous risks that accompany smallholder agricultural production in developing countries, it is plausible to expect that farmers who jointly participate in the maize and legume markets have better welfare outcomes than those who participated in either of the two markets.

### 4.2 Multinomial market participation model

We assume that farmers aim to maximize their utility *U*_*im*_ by comparing the utility provided by alternative market choices, *U*_*ik*_ such that a farmer will choose a combination of market participation alternatives, *m* over any alternative *k* if *U*_*im*_>*U*_*ik*_, *k* ≠ *m*. Following, [42], let $U_{im}^{*}$ denote the indirect utility associated with the *m*th choice, *m* = 1…4 for household *i* such that:

$$U_{im}^{*}=X_{i}B_{m}+\varepsilon_{im}$$ (1)

where *X*_*i*_ is a vector of exogenous covariates (e.g., age, education, sex, assets, and market access) and *ε*_*im*_ is the idiosyncratic unobserved stochastic component. Even though the utility of participating in the maize and legume market is not observable, we observe the decision to participate in these markets such that a farmer will choose a combination of markets *m* over any other market *k* if:

$$U=\{\begin{array}{c} 1\;if\;U_{im}^{*}>\underset{k\neq 1}{\max}\left(U_{ik}^{*}\right)\;or\;\omega_{i1}<0\\ \vdots \;\vdots \;\vdots \\ M\;if\;U_{im}^{*}>\underset{k\neq M}{\max}(U_{ik)}^{*})\;or\;\omega_{iM}<0 \end{array}\;\mathrm{for}\;\mathrm{all}\;k\neq m$$ (2)

where $\omega_{i1}=\underset{k\neq M}{\max}{(U}_{ik}^{*}-U_{im}^{*})<0.$ Assuming that *ε*_*im*_ are independent and identically Gumbel distributed, that is, under the independence of irrelevant alternatives (IIA) hypothesis, [43], then as shown by [44], Eq 1 leads to the multinomial logit model. In the multinomial logit model, the probability that a household *i* will choose market *m* can be expressed as:

$$p_{im}=Pr\left(\omega_{im}<0|X_{i}\right)=\frac{\exp\left(X_{i}B_{m}\right)}{\sum_{k\neq 1}^{j}\exp\left(X_{i}B_{k}\right)}$$ (3)

Based on the expression in Eq 3, consistent maximum likelihood estimates can be obtained [43].

#### 4.2.1 Multinomial endogenous switching regression (MESR)

In the second stage, we apply the [43] selection bias correction model to examine the relationship between each market participation choice (Table 1) and food security. This implies that households face a total of four regimes, with *m* = 1 as the reference category i.e. non-market participation. The income and food security outcome equation for each possible regime (*m*) can then be expressed as:

$$\{\begin{array}{l} \mathrm{Regime}\;1:\;y_{i1}=\beta_{1}z_{i1}+\eta_{i1}\;if\;U=1\\ \;\vdots \;\vdots \\ \mathrm{Regime}\;M:\;y_{im}=\beta_{m}z_{im}+\eta_{im}\;\mathrm{if}\;U=M \end{array}m=2,3,4$$ (4)

where *y*_*im*_ is the household income and food security of the *i*th farmer in regime *m*; *Z* represents a set of exogenous explanatory variables (e.g., household and farm-level characteristics and location variables) and *η*_*im*_ are the error terms distributed with *E* (*η*_*im*_|*X*,*z*) = 0 and $var(\eta_{im}|X,z)=\sigma_{m}^{2}$.

The outcome variables are only observed if and only if one of the possible market participation combinations is used [42, 45]. Some unobservable factors that influence the probability to participate in the market could also influence income and food security, thereby leading to non-zero covariances between the error terms of the market participation equation, *ε*_*im*_ and the outcome equation, *η*_*im*_. Therefore, the error terms in Eq (4), conditional on the sample selection criterion, have non-zero expected values, and OLS estimates will not be consistent. Consistent estimation of *β*_*m*_ requires the inclusion of the selection correction terms of the choices in Eq 4. Following [45], the selectivity term or inverse mills ratio (IMR) (which can be computed from Eq 3) can be defined as:

$$\lambda_{im}=\overset{j}{\underset{k\neq m}{\sum }}\rho_{m}\left[\frac{{\hat{p}}_{ki}\ln\left({\hat{p}}_{ki}\right)}{1-{\hat{p}}_{ki}}+\ln\left({\hat{p}}_{mi}\right)\right]$$ (5)

Where *ρ* is the correlation between *ε*_*mi*_ and *u*_*im*_. In the multinomial choice setting, there are *m*−1 selection correction terms, one for each alternative market participation combination. Following [46], we incorporate the selectivity terms (*λ*) into Eq (4) to account for selection bias such that:

$$\{\begin{array}{l} \mathrm{Regime}\;1:\;y_{1i}=\beta_{1}z_{1i}+\sigma_{1}{\hat{\lambda }}_{i1}+\nu_{i1}\;if\;U=1\\ \;\vdots \;\vdots \\ \mathrm{Regime}\;M:\;y_{im}=\beta_{m}z_{im}+\sigma_{m}{\hat{\lambda }}_{im}+\nu_{im}\;if\;U=M \end{array}m=2,3,4$$ (6)

where *σ* is the covariance between *ε*_*im*_ and *u*_*im*_; and *v*_*im*_ is the error term with an expected value of zero.

Although in principle, the parameters of the model can be identified using the non-linearities generated through the model (i.e., the IMR), we use exclusion restrictions or instruments for a more robust identification [47]. To achieve this, we need an instrumental variable (IV) correlated with the decision to participate in the market but does not determine income and food security, conditional on participation. We use the average number of motorcycles and bicycles (hereinafter “transport equipment”) owned by households living in the same ward as the farmer himself/herself. A ward is an administrative structure or local authority area for a single town or portion of a bigger town (urban wards) which is smaller than a district. Rural wards are composed of several villages.

We constructed this instrument following [9, 11]. First, we counted the number of transport equipment owned by sample households in each ward but excluding the household in question. After that, we divided this number by the number of sample households in each ward, giving us a proportion of households with transport equipment in the ward. Averaging the number of transport equipment in a ward as opposed to individual ownership ensures that the instrument is not directly correlated with our household income and food security variables. The number of transport equipment in the ward implies better market access because in developing countries, most of the local roads are not paved and public transport may not exist; hence, owners of transport equipment often offer transport services to other households living in the same area [9, 11]. Studies that have used similar instruments include [9, 11, 48, 49]. Coupled with this intuitive justification, we also conducted a test to assess the suitability of this instrument. We followed [42] in performing a falsification test: if a variable is a valid selection instrument, it will affect the market participation decision, but it will not affect the income and food security among farm households that did not participate in the markets. Results of the test confirm that in all cases that our instrument is significant in the market participation equations (Table 3) but not in the income and food security equation among the non-market participants (A1 Table in S1 Appendix). Although our constructed instrument satisfies all the post estimation tests, including using a rich cross-sectional dataset, our instrument can still be contested. For example, the exogeneity condition might not be satisfied should households with relatively higher welfare be more likely to reside in wards where neighboring households own a larger number of motorbikes and bicycles. While the consistency of the results across the two methods we use in the paper support evidence of impact, the results should still be interpreted with some caution.

#### 4.2.2 Estimation of average treatment effects on the treated (ATT)

In the present study, of significant interest is the effect of market participation on income and food security outcomes. Specifically, we use the MESR framework mentioned above to derive the expected actual and counterfactual income and food security outcomes. Following [45] and [47] the expected food security under the actual scenario for each choice is computed as follows:

$$E\left(y_{im}|U=m,z_{im},{\hat{\lambda }}_{im}\right)=\beta_{m}z_{im}+\sigma_{m}{\hat{\lambda }}_{im}$$ (7A)

The expected food security value of the same farmer had he/she chosen not to participate in any market (i.e. the counterfactual) is given as:

$$E(y_{1i}|U=m,z_{im},{\hat{\lambda }}_{im})=\beta_{1}z_{im}+\sigma_{1}{\hat{\lambda }}_{im}$$ (7B)

Thus, the difference in expected outcomes between Eqs (7A) and (7B) is the unbiased average treatment effect on the treated (ATT)–which measure the impact of market participation for the households who participated in the market–and this is given as:

$$\begin{array}{l} ATT=E(y_{im}|U=m,z_{im},{\hat{\lambda }}_{im})-E(y_{1im}|U=m,z_{im},{\hat{\lambda }}_{im})\\ \;=z_{im}\left(\beta_{m}-\beta_{1}\right)+{\hat{\lambda }}_{im}\left(\sigma_{m}-\sigma_{1}\right) \end{array}$$ (8)

This approach postulates that unobserved factors have differential effects on participants and non-market participants, hence taking the differences in effects, i.e. *σ*_*m*_−*σ*_1_, while holding ${\hat{\lambda }}_{im}$ constant ensures that the effects of unobserved factors are cancelled out [50].

### 4.3 Multivalued inverse probability weighted regression adjustment

The MESR is strictly dependent on the availability of an instrument satisfying several econometric requirements for exogeneity, validity, and strength for the identification of the model, however finding an instrument with these characteristics in practice is difficult. Even though evidence shows that the instrument we have identified in Section 4.2.1 satisfies all the required conditions, there is a possibility that the model may still not be properly identified and, as such, we complement the MESR model with the MIPWRA model, which in any case only accounts for observed characteristics. This algorithm uses the inverse of the estimated treatment probability weights to estimate missing data-corrected regression coefficients that are subsequently used to produce robust estimates of ATT.

The estimation of the model proceeds in two steps. In the first step, the parameters of the propensity score model (market participation or treatment model) are estimated using a multinomial logit model, after which the inverse probability of treatment weights are calculated for each level of treatment. In the second step, using the estimated weights, the income and food security models are fitted by a weighted regression for each treatment level, and treatment-specific predicted outcomes for each household are obtained using the estimated coefficients from this weighted regression model [51]. The model is finally estimated using generalized methods of moments (GMM) in one step which has the advantage of automatically accounting for the estimation error from the estimated propensity scores when deriving the standard errors.

For the sake of brevity, we are not going to present all the details of the model, but [51–54] give details on the derivation of the MIPWRA model, while [55] describe the theory for semiparametric estimators. We can define the average treatment for the households who participated in the maize and legume markets (ATT) as:

$$ATT_{\tilde{ti},\vec{t}}=E\left\{\left(y_{\tilde{ti}}-y_{1i}\right)|t=\vec{t}\right\}$$ (9)

Where *y*_*ti*_ is the potential outcome (income and food security) that household *i* would obtain given treatment-level *t*. The *t*, in this case, is analogous to *m* above where *t* = 1…4 for household *i*. In the multivalued treatment case, the ATT requires three different treatment levels: $\tilde{t}$ defines the treatment level of the treated potential outcome; 0 is the treatment level of the control potential outcome, and; $t=\vec{t}$ restricts the expectation to include only those individuals who receive treatment level $\vec{t}$.

As with all models based on observed characteristics, the MIPWRA relies mainly on two assumptions for the results to be valid. The first assumption is the conditional independence assumption (CIA), which postulates that the treatment assignment is essentially randomized conditional on observables. This assumption implies that the potential outcome distributions are independent of the treatment level. Therefore, it rules out that some unobservable factor correlated with treatment assignment affects the potential-outcome distributions [56]. Unfortunately, there no formal tests to test whether this assumption holds in our case. The second assumption is the overlap assumption which ensures that each household could receive any treatment level. In the subsequent sections, we test the overlap assumption using density distributions to assess whether balancing was achieved using the MIPWRA model.

## 5. Results and discussion

### 5.1 Descriptive statistics

Table 2 below provides descriptive statistics of the variables used in the study. Results indicate that, on average, households spent about Tsh 363,708 in the year preceding the survey on food and non-food items, split almost equally among the two categories.

**Table 2:** Descriptive statistics of selected variables.

Variables	Description		
		Mean	SD
*Dependent variables*			
Total household expenditure	Total household expenditure per capita in MWK/TSh	363,708	318,401
Food expenditure	Food consumption expenditure per capita in MWK/TSh	183,332	159,829
HDDS	Household dietary diversity scores (number)	7.561	2.040
Months insecure	Number of months household in food insecure (number)	0.483	1.383
HFIAS	Household food insecurity access scale (number)	0.8111	0.382
*Independent variables*			
Sex of head	1 = male- headed household.	0.864	0.343
Primary school	1 = Proportion of household heads who completed primary school education.	0.670	0.470
Number of adults	Number of adults from 15–59 years old	2.930	1.636
Cultivated land	Total land cultivated in hectares	2.426	5.535
Square of cultivated land	The square of total cultivated land cultivated in hectares	36.49	542.8
Months lived	The number of months the household head lived with the household in the past year (Tanzania)	2.607	2.335
Livestock ownership	Livestock ownership measured in Tropical Livestock Units (TLU)	3.773	8.172
Non-farm income	1 = if had access to off-farm income	0.295	0.457
Implement index	Agricultural implement index	-1.74e-09	1.735
Credit	1 = received credit	0.238	0.426
Mobile phone	1 = owns mobile phone	0.789	0.408
Organic fertilizer	1 = applied organic fertilizer	0.557	0.497
Intercropping	1 = practiced intercropping	0.980	0.139
Drought	1 = experienced a drought shock in the past five years	0.236	0.425
Crop pests	1 = experienced crop pests and diseases in the past five years	0.231	0.422
Main market	The average number of main market participants	3.115	3.591
Market accessibility	Travel time required to reach the nearest urban centre (minutes)	75.540	2.429
Distance asphalt road	Distance from the house using to the nearest asphalt or tarmac road (minutes)	8.264	8.515
Transport equipment	Percentage of motorcycles and bicycles in a ward	63.093	14.464
Number of observations		809	

Note: The average official exchange rates in the year the surveys were conducted: 1US$ = Tsh 1653.23 (https://data.worldbank.org/indicator/PA.NUS.FCRF?locations=TZ).

The average HDDS is eight -out of 12- signaling a relatively high diversity overall, and it is in line with the value found by [57]. On average, households indicate that they have experienced about 0.5 months of food insecurity over the past 12 months.

Over 85% of the households are headed by males. A typical household has about three adults in the working age category -between 15 and 59 years old-, a proxy of labor availability in the household for production and marketing activities [58, 59]. On average, households cultivate 2.4 ha and own a herd size of about three tropical livestock units (TLU). About one in four households reports not having access to credit, which is crucial to lessen food risks related to uncertain cash flow and food crop prices [35].

The percentage of households who apply organic fertilizers and practice intercropping is 56% and 98%, respectively, with the use and adoption of such technologies affecting maize and legume productivity that in turn positively affect famers’ marketable surplus [38]. The latter can be effectively translated into substantial income benefits given the relatively high accessibility to markets. On average, farmers in our sample need to travel just 8 minutes to access a tarred road-. The market access variable is proxied by the travel time required to reach the nearest urban center, defined as a contiguous area with 1,500 or more inhabitants per square kilometer or as a majority of built-up land cover coincident with a population center of at least 50,000 inhabitants [60]. The relatively high accessibility reported is also a function of ownership of motorbikes or bicycles, given that about 63% of the households in each ward owns either a motorbike or bicycle.

**Fig 1:**
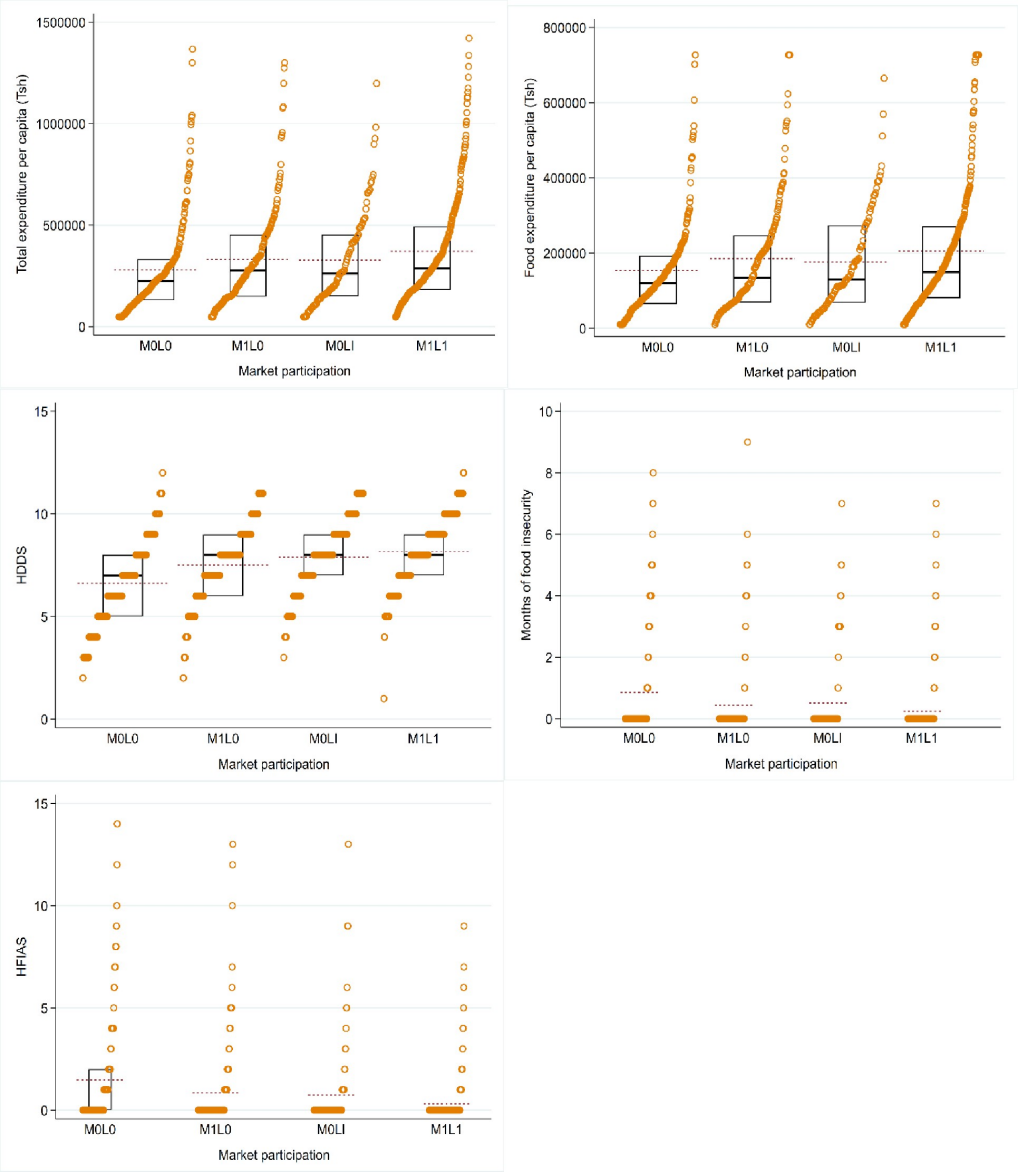
Distribution of outcome variables by market participation.

To gain an initial insight into the relationship between market participation and food security, Fig 1 presents the data distribution using strip plots. The plots show the distributions of income and food security by each market participation category with the associated cumulative probabilities. Farmers who participate in either maize or legume market report higher income and better food security than non-participants. Moreover, income and food security distribution functions for market participants dominate those for non-participants. Overall, households that participate in joint maize and legume markets report the highest food security outcomes. A2 Table in S1 Appendix consistently shows a statistically significant difference between the mean outcomes of the joint market participants and the other market participations. However, the average number of months of food insecurity and HFIAS were only marginally lower for joint maize and legume participants relative to non-market participants. However, these descriptive findings are only bivariate unconditional relationships, since we did not control for other characteristics that might affect the outcome variables, which we will do in our multinomial regression framework.

### 5.2 Determinants and impact of market participation on income and food security

#### 5.2.1 Determinants of maize and legume market participation

Table 3 presents the parameter estimates from the multinomial logit model described in Section 4, i.e., the first stage results of the MESR model. The standard errors reported are corrected for intra-cluster correlation at the village level, given the sampling design and the expected correlation of the characteristics across households within each village. Consistently with our *a priori* expectations, results show that education, amount of time the household head spent within the household -a proxy for labor availability-, amount of cultivated land, and the index of ownership of agricultural implements are all positively related to the joint participation in maize and legume markets. Labor, land, and assets are the factors of production enabling farmers to produce a marketable surplus [36, 61]. Farmers who obtained credit show a higher propensity to participate in maize and legume markets, as the literature also finds -e.g., [36] for Kenya-. Surprisingly, ownership of mobile phones traditionally and empirically associated with increased market participation [5, 62] seems to be negatively related to joint participation in maize and legume markets, likely due to the lack of use of mobile phones in trade business that occurs on the spot market and with random traders instead of by phone.

**Table 3:** Multinomial selection model parameter estimates.

Variable	Maize only	Legumes only	Joint maize and legumes
Sex of the household head	-0.011	0.110	0.327
	(0.334)	(0.411)	(0.298)
Completed primary school	0.271	0.043	0.454**
	(0.257)	(0.230)	(0.226)
Number of adults	0.031	-0.138**	-0.020
	(0.055)	(0.065)	(0.076)
Cultivated land	-0.052	0.063	0.296***
	(0.182)	(0.232)	(0.112)
Square of total cultivated land	-0.006	-0.003	-0.002***
	(0.016)	(0.015)	(0.001)
Months lived	0.074	-0.091	0.141***
	(0.059)	(0.095)	(0.051)
Livestock ownership	0.012**	0.018*	0.001
	(0.006)	(0.009)	(0.013)
Access to non-farm income	0.265	0.375	0.162
	(0.342)	(0.242)	(0.279)
Implement index	0.265***	0.042	0.187**
	(0.068)	(0.122)	(0.077)
Received credit	0.407	0.359	0.870***
	(0.258)	(0.333)	(0.262)
Mobile phone	-0.012	-0.019	-0.063*
	(0.022)	(0.038)	(0.033)
Treated group	-0.528	-0.183	0.229
	(0.343)	(0.347)	(0.295)
Applied organic fertilizer	0.089	0.050	0.471*
	(0.320)	(0.298)	(0.268)
Practiced intercropping	2.344***	0.714	1.464
	(0.864)	(1.077)	(1.369)
Drought shock	-1.079***	-0.622	-0.600*
	(0.315)	(0.386)	(0.327)
Crop pests shock	0.339	-0.409	-0.036
	(0.223)	(0.443)	(0.312)
Sold to main market	-0.090**	0.051	0.055
	(0.039)	(0.095)	(0.068)
Distance nearest asphalt road	-0.034**	-0.016	-0.031**
	(0.017)	(0.022)	(0.015)
Access to markets	-0.000	-0.016	-0.010**
	(0.003)	(0.011)	(0.005)
Percentage of transport equipment	0.026**	0.035	0.074***
	(0.012)	(0.035)	(0.028)
Manyara region	0.578	1.109	3.844***
	(0.493)	(0.836)	(0.925)
Constant	-3.433**	-1.729	-5.087**
	(1.577)	(1.676)	(2.221)

Note: Standard errors corrected for intra-cluster correlation in parenthesis.

* p<0.10

** p<0.05

*** p<0.001. The base category is market non-participation.

The use of organic fertilizers and intercropping is usually positively correlated with marketable surplus given they enhance maize and legume productivity [63]. Results in Table 3 show that the adoption of organic fertilizers and intercropping indeed increase participation in the legume market only and legume and maize markets, in line with [64] who find that legume sellers are more likely to practice intercropping in Malawi. In line with expectations, the occurrence of droughts reduces market participation via lower production of maize and legumes.

Distance to a tarred road and urban center are negatively associated with maize and legume market participation because of the increased transaction costs, with this finding consistent across market participation options. As expected, the prevalence of transportation durable assets in a ward affects the likelihood of participating in all markets, likely due to the reduction in transportation costs and enhanced opportunities in more distant and profitable markets [11, 36, 61].

Looking at the geographical heterogeneity, farmers in the Manyara region are more likely to participate in maize and legume markets than those in the Dodoma region. The former is considered a high agricultural potential area with good climatic conditions, ideal for maize and legume cultivation unlike the latter. On the other hand, Dodoma is a semi-arid region also prone to soil erosion and flooding, and hence farmers can attain only a relatively low marketable surplus. This strongly negative relationship between climate risk and market exposure is also consistent with [61], who find that areas with higher climatic risks are associated with less commercialization in Mozambique.

### 5.3 Impact of maize and legume market participation on income and food security

#### 5.3.1 Results of the multinomial endogenous switching regression model (MESR)

Table 4 displays the average effect of maize and legume market participation on household welfare indicators based on the estimation of Eq 8. Results show that participation in single and joint markets leads to a statistically significant increase in many of the outcome variables considered. Total household expenditure increases due to participation in maize-only market is 12% higher than that of market non-participation. More importantly, joint participation in maize and legume markets results in a 31% increase in household expenditure. A similar trend is observed for food expenditure, with the joint market participation showing the highest percentage increase of 24% compared to the counterfactual group. Previous studies [e.g., 65–67] show that purchased foods contribute substantially to total calorie consumption in most developing countries, even among subsistence farmers.

**Table 4:** Impact of maize and legume market participation on income and food security using the MESR.

Market participation status	Outcome	Total household expenditure (Tsh)	Food expenditure (Tsh)	HDDS	Months of food insecurity	HFIAS
Maize market participants	Participants	346000	185000	7.511	0.428	0.851
	Non-participants	308000	160000	7.162	0.699	1.174
	*ATT*	38403.35*** (8162.633)	25327.68*** (4676.519)	0.348*** (0.069)	-0.200*** (0.061)	-0.323*** (0.111)
	% change in outcome	12%	16%	5%	-27%	-28%
Legume market participants	Participants	327000	176000	7.889	0.511	0.745
	Non-participants	308000	164000	7.526	0.499	1.27
	*ATT*	19756.56 (12684.92)	11856.57* (6716.816)	0.348*** (0.069)	0.012 (0.093)	-0.526*** (0.146)
	% change in outcome	6%	7%	5%	-2%	-41%
Maize and legume market participants	Participants	438000	206000	8.156	0.241	0.354
	Non-participants	334000	165000	7.72	0.409	1.025
	*ATT*	104000*** (4291.799)	40224.95*** (4291.799)	0.437*** (0.143)	-0.168*** (0.041)	-0.67*** (0.066)
	% change in outcome	31%	24%	6%	-41%	-66%

Note: Standard errors corrected for intra-cluster correlation in parenthesis.

* p<0.10

** p<0.05

*** p<0.001. The base category is market non-participation.

Compared with counterfactuals, for farmers participating in maize-only markets, HDDS increases by 5% while the number of months of food insecurity reduces by 27%. Participation in the legume-only market is also associated with a 5% gain in HDDS. Similarly, participation in the maize market-only reduces the HFIAS scores by 28%. For all our food security indicators, the highest gains are associated with joint maize and legume market participation. For instance, HDDS increases by 6% due to joint participation in the two markets, a higher effect than participation in either maize-only or legume-only market. Likewise, joint participation in maize and legume markets reduces the number of months of food insecurity and HFIAS by 41% and 66% respectively. Interestingly, except for the HFIAS, the positive effects of maize-only market participation are generally higher than those accruing from legume-only market participation, likely due to the dominance of maize as a staple food in the Tanzanian diet.

#### 5.3.2 Results of the multinomial inverse probability weighted regression model (MIPWRA)

We also estimate the ATT using the MIPWRA model (based on Eq 9) as a robustness check for our MESR results. ATT results are valid if they are drawn from observationally identical groups according to the propensity score, which synthetically summarizes the likelihood of the samples under analysis being comparable. A1 Fig in S1 Appendix shows that the overlap assumption of our groups is indeed satisfied after propensity score reweighting. Parameter estimates associated with treatment (first stage) and outcome (second stage) equation models are presented in Table 3 and A5 Table in S1 Appendix, respectively.

After controlling for observed heterogeneity only, ATT estimates in Table 5 indicate that joint market participation in maize and legume markets is associated with the largest gains in income and food expenditure. Consistent with estimates in Table 4, MIPWRA results show that participation in maize-only markets increases HDDS by 7%, while it reduces the number of months of food insecurity and HFIAS by 40% and 34%, respectively, compared with market non-participants. However, joint participation in maize and legume markets increases HDDS by 12% and reduces the number of food-insecure months and HFIAS by 63% and 85%, respectively. These results suggest that additional benefits are obtained when farmers simultaneously participate in maize and legume markets compared with single-commodity market participation. MIPWRA estimates are quantitatively slightly larger than those from the MESR model, owing to the underlying lack of the former in controlling for unobserved heterogeneity. Nevertheless, the similar magnitude of MIPWRA and MESR parameter estimates provides overall confidence in our multivariate regression framework specification.

**Table 5:** Impact of maize and legume market participation on income and food security using MIPWRA.

Market participation status	Outcome	Total household expenditure (Tsh)	Food expenditure (Tsh)	HDDS	Months of food insecurity	HFIAS
Maize market participants	Participants	260667.2582	131531.284	7.513	0.428	0.851
	Non-participants	230268.201	111413.078	7.021	0.711	1.288
	*ATT*	30399.057** (15509.72)	20118.205** (8347.80)	0.492*** (0.171)	-0.283* (0.148)	-0.437* (0.230)
	% change in outcome	13%	18%	7%	-40%	-34%
Legume market participants	Participants	237993.823	114462.21	7.660	0.349	0.702
	Non-participants	230268.201	111413.078	7.021	0.711	1.288
	*ATT*	7725.622 (16437.49)	3049.131 (14519.67)	0.639* (0.379)	-0.362 (0.289)	-0.586* (0.292)
	% change in outcome	3%	3%	9%	-51%	-45%
Maize and legume market participants	Participants	301040.474	143630.599	7.875	0.265	0.194
	Non-participants	230268.20	111413.078	7.021	0.711	1.288
	*ATT*	70772.273*** (15797.38	32217.521*** (9284.588)	0.854*** (0.289)	-0.446*** (0.170)	-0.67*** (0.066)
	% change in outcome	31%	29%	12%	-63%	-85%

Note: Standard errors corrected for intra-cluster correlation in parenthesis.

* p<0.10

** p<0.05

*** p<0.001. The base category.

## 6. Conclusions and policy implications

In this study, we examine the effect of market participation on household income and food security in three districts across two regions in Tanzania using socio-agro-economic household survey data. Univariate descriptive statistics point towards a statistically significant difference in the average characteristics of joint maize and legume participants *vis-à-vis* participants in single maize or legume markets. To control for possible confounding factors and likely endogeneity intervening in the relationship between market participation and welfare outcomes in an econometric framework, we employ the multinomial endogenous switching regression model, complemented with the multivalued inverse probability weighted regression model, which is doubly-robust allowing one of the equations -treatment status or outcome prediction- to be misspecified.

Results from the first stage regression reveal that the likelihood of contemporaneous participation in the maize and legume markets increases with education, social capital, ownership of land, productive farm assets, adoption of improved technologies, and ownership of transportation equipment. Market participation, however, decreases with the occurrence of droughts. Farm productive assets seem to be crucial in increasing maize and legume productivity; hence provision of credit can enable smallholder farmers to relax liquidity constraints hampering ownership and use of these implements for more productive farming. Similarly, encouraging the adoption of intercropping and organic fertilizer application is vital in increasing maize and legume marketable surplus, thereby increasing market participation.

Our results also show that participating in the maize market only increased household income by 12% and food expenditure by 16%, other things being equal. Participation in legume markets leads to quantitatively comparable effects. However, across all the outcome variables considered, our results suggest that farmers who jointly participate in maize and legume markets attain higher income and improved food security than those who participate in either maize or legume market. Smallholder farmers who jointly participate in the two markets spend as much as 24% of their income on food, attained a more diversified diet, and are subject to fewer months of food insecurity over a year.

Two main policy implications can be drawn from this study. First, improving the functioning of agricultural markets through the facilitation of market access and reduction in transaction costs is vital for smallholder farmers to fully reap the welfare benefits of market participation in rural Tanzania, as is the case in many similar rural settings in Africa south of the Sahara. Despite the recent construction of a promising development corridor in southern Tanzania, the country is still plagued with poor road infrastructure that hinders farmers from accessing profitable maize and legume markets available in urban centers locking them in poverty. Hence, development programmes and policies aimed at reducing transport and transactions costs, as well as curbing travel time to profitable markets through road network improvement in rural communities are necessary for smallholder farmers to prevent their market and, hence, economic isolation.

Second, our findings suggest the need to support policy measures that promote the combined production and marketing of maize and legumes given their strong poverty-alleviation potential shown in our study. These interventions should follow a diversification strategy enhancing maize-legume intercropping and rotation, as opposed to a specialization strategy focusing on one single crop, given the empirical evidence pointing to disproportionately higher income and food security benefits of joint maize and legume market participation. This strategy would also smooth the seasonality of consumption given the different growing periods of the two crops. In areas where vulnerability to climate change and shocks is relatively high, as in most Africa south of the Sahara, participation in multiple-commodity markets would also provide a potential hedge strategy against the risk of price shocks, strengthening household resilience and welfare conditions.

## Supporting information

S1 Appendix (DOCX)
