# Supplementary material for: Welfare impacts of smallholder farmers’ participation in multiple output markets: Empirical evidence from Tanzania
Source: PLoS One. 2021 May 6;16(5):e0250848. doi: 10.1371/journal.pone.0250848 (PMC8101961; doi:10.1371/journal.pone.0250848)
Supplement: S1 Appendix — (DOCX) [file pone.0250848.s001.docx]

**S1 Appendix.**

A1 Table: Test on the validity of the instruments

| Outcome variables | Total household expenditure | Total household expenditure | HDDS | Months of insecurity | HFIAS |
| --- | --- | --- | --- | --- | --- |
| Sex of the household head | 23,549.528 | 1,272.220 | 0.182 | -0.285* | -0.626*** |
|  | (19,234.098) | (10,141.825) | (0.215) | (0.163) | (0.202) |
| Completed primary school | -36,702.111 | -19,848.321 | 0.004 | 0.019 | -0.177 |
|  | (33,511.552) | (14,113.367) | (0.138) | (0.100) | (0.152) |
| Number of adults | 4,084.823 | -2,289.943 | 0.058** | -0.044* | 0.007 |
|  | (4,567.431) | (2,152.636) | (0.029) | (0.025) | (0.046) |
| Cultivated land | 29,132.846*** | 10,080.063*** | 0.060 | -0.019 | 0.017 |
|  | (7,196.626) | (2,946.075) | (0.038) | (0.020) | (0.047) |
| Square of total cultivated land | -234.554*** | -73.273*** | -0.001* | 0.000 | -0.000 |
|  | (53.857) | (20.188) | (0.000) | (0.000) | (0.000) |
| Months lived | 64,140.866*** | 30,399.407*** | -0.050** | -0.056*** | -0.066 |
|  | (4,713.462) | (2,786.343) | (0.024) | (0.018) | (0.043) |
| Livestock ownership | 512.022 | -1,769.662*** | -0.007 | -0.011*** | -0.015*** |
|  | (3,049.540) | (523.860) | (0.008) | (0.003) | (0.006) |
| Access to non-farm income | 100,582.206*** | 37,201.025*** | 0.402*** | -0.217*** | -0.218 |
|  | (20,795.864) | (12,106.643) | (0.095) | (0.083) | (0.133) |
| Agricultural implement index | 17,493.660* | 3,555.320 | 0.209*** | -0.093** | -0.149** |
|  | (8,998.550) | (3,967.343) | (0.036) | (0.046) | (0.068) |
| Received credit | 60,308.928** | 36,850.983*** | 0.183 | 0.235* | 0.094 |
|  | (26,260.989) | (11,195.187) | (0.128) | (0.129) | (0.115) |
| Mobile phone | 1,233.571 | 1,204.479** | 0.021 | -0.011 | -0.008 |
|  | (1,549.529) | (561.260) | (0.014) | (0.013) | (0.024) |
| Treated group | -8,840.077 | -15,119.872 | 0.315* | 0.077 | 0.055 |
|  | (34,739.117) | (13,667.912) | (0.172) | (0.103) | (0.184) |
| Applied organic fertilizer | -3,031.149 | -7,684.683 | -0.011 | -0.124 | -0.105 |
|  | (26,486.728) | (9,514.923) | (0.134) | (0.111) | (0.161) |
| Practiced intercropping | -76,771.188 | -24,457.861 | -0.114 | 0.017 | 0.274 |
|  | (72,570.796) | (22,852.785) | (0.457) | (0.217) | (0.264) |
| Drought shock | -43,919.279* | -28,996.977** | -0.447** | -0.014 | -0.031 |
|  | (23,270.911) | (12,803.631) | (0.227) | (0.147) | (0.284) |
| Crop pests shock | 34,409.674 | 20,942.873* | 0.050 | 0.095 | 0.307*** |
|  | (22,691.327) | (11,978.794) | (0.159) | (0.108) | (0.115) |
| Sold to main market | 8,564.523** | 6,040.258*** | -0.008 | -0.010 | -0.012 |
|  | (3,554.814) | (1,710.783) | (0.026) | (0.018) | (0.029) |
| Distance nearest asphalt road | -2,913.433 | -1,807.340* | -0.021** | 0.006 | 0.016 |
|  | (1,775.621) | (947.576) | (0.011) | (0.005) | (0.012) |
| Access to markets | -43.300 | -56.398 | -0.004 | -0.001 | -0.004 |
|  | (189.611) | (73.148) | (0.003) | (0.001) | (0.003) |
| Percentage of transport equipment | -285.434 | -798.266 | -0.014 | 0.006 | 0.000 |
|  | (2,043.466) | (836.386) | (0.013) | (0.005) | (0.007) |
| Manyara region | 61,995.965 | -9,285.999 | 1.063** | -0.663* | -1.463** |
|  | (62,429.211) | (25,400.733) | (0.414) | (0.380) | (0.622) |
| Constant | 35,431.332 | 85,581.142* | 6.213*** | 2.181** | 3.395** |
|  | (123,794.153) | (49,216.894) | (1.153) | (0.926) | (1.590) |

Notes: Standard errors corrected for intra-cluster correlation in parenthesis. * p<0.10, ** p<0.05, *** p<0.001.

A2 Table: Market participation by income and food security

| Market participation choice | Total household  expenditure | Food expenditure | HDDS | Months of food insecurity | HFIAS |
| --- | --- | --- | --- | --- | --- |
| Non-participants | 283078.10***  (16128.54) | 153044.50***  (8878.11) | 6.632***  (0.14) | 0.864***  (0.12) | 0.311***  (0.03) |
| Maize only | 348474***  (22435.39) | 182352***  (12121.89) | 7.567***  (0.15) | 0.439  (0.11) | 0.178  (0.03) |
| Legumes only | 327391.8 ***  (25482.11) | 176272.7***  (14626.82) | 7.889***  (0.2) | 0.511  (0.14) | 0.144  (0.04) |
| Joint maize and legumes | 437611.30  (20909.28) | 203669.60  (9821.05) | 8.172  (0.1) | 0.243  (0.05) | 0.095  (0.02) |

Note: Standard errors corrected for intra-cluster correlation in parenthesis. * p<0.10, ** p<0.05, *** p<0.001. The base category is market non-participation.

A3 Table: Determinants of income and food security (second stage results of the MESR model)

| Variable | Total expenditure | | | | Food expenditure | | | |
| --- | --- | --- | --- | --- | --- | --- | --- | --- |
|  | M_0_ L_0_ | M_1_ L_0_ | M_0_ L_1_ | M_1_ L_1_ | M_0_ L_0_ | M_1_ L_0_ | M_0_ L_1_ | M_1_ L_1_ |
| Sex of head | 70653.510  (47331.22) | 60852.870  (71994.69) | -67336.060  (174116.5) | 88426.820 (88426.82) | 28138.52 (25982.93) | -1323.814 (41018.01) | -27683.27 (93680.29) | 17009.6 (36979.55) |
| Primary school | -11118.620 (32428.94) | -46328.330 (72576.05) | -102201 (108425.7) | 67541.080 (67541.08) | -2137.812 (19312.92) | -35635.79 (40896.92) | -2347.58 (73692.34) | -11535.96 (25880.63) |
| Number of adults | 10880.330 (14797.15) | -14552.730 (20107.99) | -3182.664 (50597.44) | 25368.430 (25368.43) | 6625.422 (8118.202) | -11366.79 (11275.26) | 9659.327 (35038.14) | 7587.566 (10420.62) |
| Cultivated land | 25352.950 (27384) | -24499.710  (43047.36) | 25587.66 (106431.50) | 34064.600 (34064.60) | -309.721  (12200.71) | -26168.22 (28470.15) | -6025.024 (61097.79) | 9838.549 (13450.9) |
| Square of cultivated land | -515.275 (1533.465) | 4457.915 (6063.967) | 2809.482  (23578) | 1372.774 (1372.774) | 347.473 (755.4526) | 4602.299 (3334.758) | -235.218  (12085.39) | -43.459  (514.1046) |
| Months lived | 63314.350*** (11694.52) | 54662.590** (21197.97) | 53426.15 (60570.34) | 17681.25*** (17681.250) | 29902.44 (9497.752) | 25294.15**  (10182.91) | 42981.16 (41068.08) | 45202.56*** (7946.845) |
| Livestock ownership | 4515.903 (6269.917) | 2540.004 (10219.27) | -14071.42 (12511.68) | 8032.618 (8032.618) | -1044.328 (1724.02) | -1058.438 (5431.157) | -7718.983 (8167.069) | -3279.408 (2756.261) |
| Non-farm income | 71006.610* (39683.31) | 150297.900 (75977.37) | 99228.84 (143839.6) | 78549.840 (78549.84) | 54289 (23605.13) | 67516.04*  (38622.29) | 9192.019 (79010.44) | 18696.14 (29910.5) |
| Implement index | 9356.834 (23737.66) | 40741.490 (28600.19) | 34797.24 (58738.54) | 31203.070 (31203.070) | 4214.503 (10881.64) | 18926.03 (14785.74) | 51261.38 (39057.48) | 12316.84 (13155.68) |
| Received credit | 36509.070 (48052.43) | 120924.200 (89367.93) | 54433.48 (149490.8) | 68040.63 (68040.630) | 18696.25 (26724.56) | 79231.65 (52753.38) | 54419.57 (95335.23) | 42315.51 (26656.96) |
| Mobile phones | 1264.093 (1246.537) | 3595.752 (2769.09) | -57.79651 (6358.231) | 4876.270 (4876.270) | 699.409 (638.2554) | 619.601  (1330.07) | -1481.258 (5238.928) | 753.297  (1612.596) |
| Treatment group | 21415.08 (76538.48) | -95940.580  (121940) | 3762.08 (192829.1) | 93486.720 (93486.72) | -14599.88 (37186.72) | -29270.86 (58018.58) | -49870.84 (104036.5) | -10817.35 (37853.87) |
| Organic fertilizer | -18844.320 (36734.75) | -6871.715 (70087.78) | 11744.82 (82201.1) | 58220.97 (58220.97) | -25726.12 (21885.44) | -652.515  (40892.43) | 4756.766 (60479.07) | 30637.87 (25509.36) |
| Intercropping | -17135.640 (128650.40) | -244070.700  (270282.1) | -275711.5 (450595.3) | 384881.4 (384881.4) | -8807.124 (63615.77) | -84535.78 (114036) | 142861.3 (248944.1) | 13385.64 (165482.8) |
| Drought shock | -40162.60 (78373.57) | -169640.500  (140614.7) | 113061.8 (225700.6) | 99254.98 (99254.98) | -34475.24 (35987.98) | -80316.32 (63416.83) | -57777.29 (104612.7) | -39437.96 (41117.01) |
| Crop pests shock | 60618.08 (60426.68) | 83103.820  (84938.03) | 71942.8 (215295.7) | 77684.07 (77684.07) | 18738.58 (31118.86) | 35745.69 (43993.38) | 146617.2 (129796.8) | 51208.84 (33650) |
| Main market | 6637.314 (11641.03) | -16264.14 (23215.26) | 21211.69 (30115.94) | 14366.57 (14366.57) | 4454.342 (6425.996) | -7803.008 (8896.337) | -4497.034 (18256.04) | 922.114  (7103.276) |
| Distance asphalt road | -835.362 (2840.904) | -1545.134 (5310.265) | 509.0104 (10055.77) | 4780.557* (4780.557) | -903.6447 (1163.334) | -399.613 (2908.916) | -7290.295 (6616.143) | -4568.619** (1885.857) |
| Market accessibility | -159.147  (409.935) | 48.335  (873.7181) | -145.0389 (4207.633) | 1562.751 (1562.751) | -189.6196 (221.4409) | 55.904 (550.417) | 1802.396 (2622.201) | 635.671  (586.884) |
| Manyara region | 62225.720  (80094.90) | -38258.7 (133570.4) | 57329.18 (304213.7) | 145834.9 (145834.9) | 22585.83 (42614.54) | -12081.25 (66825.28) | -49874.37 (189991.2) | 23541.42 (58232.56) |
| Constant | -91070.650 (155482.6) | -124063.300 (577807.2) | 622557.7 (812822.6) | 665721.4 (665721.4) | 49913.37 (87025.28) | 121010.7 (265006.9) | 400797.2 (611960.4) | -152863.1 (247024.2) |

Note: Standard errors corrected for intra-cluster correlation in parenthesis. * p<0.10, ** p<0.05, *** p<0.001. The base category (M_0_L_0_) is market non-participation; M_1_L_0_: only maize market participation; M_0_L_1_: only legume market participation; M_1_L_1_: joint maize and legume markets participation.

A4 Table: Unconditional average treatment effects from the MESR model

| Market Participation choice | Total household expenditure | Food expenditure | HDDS | Months of insecurity | HFIAS |
| --- | --- | --- | --- | --- | --- |
| Maize only | 116376.20***  (31039.72) | 78374***  (25481.65) | 0.523***  (0.049) | -0.355***  (0.033) | -1.081***  (0.326) |
| Legumes only | 30699.41*  (18448.25) | -9588.88  (7304.40) | 0.257***  (0.082) | -0.043  (0.136) | 0.030  (0.337) |
| Joint maize and legumes | 103030.4***  (5777.794) | 42538.32***  (2543.87) | 0.837***  (0. 059) | -0.360***  (0.026) | -0.957***  (0.044) |

Note: Standard errors corrected for intra-cluster correlation in parenthesis. * p<0.10, ** p<0.05, *** p<0.001. The base category is market non-participation.

A5 Table: Determinants of income and food security (second stage results of the MIPWRA model)

| Variables | Log total expenditure | | | | Log food expenditure | | | | HDDS | | | |
| --- | --- | --- | --- | --- | --- | --- | --- | --- | --- | --- | --- | --- |
|  | M_0_ L_0_ | M_1_ L_0_ | M_0_ L_1_ | M_1_ L_1_ | M_0_ L_0_ | M_1_ L_0_ | M_0_ L_1_ | M_1_ L_1_ | M_0_ L_0_ | M_1_ L_0_ | M_0_ L_1_ | M_1_ L_1_ |
| Sex of the household head | 0.357*** | 0.304** | -0.316* | 0.179 | 0.381** | -0.074 | -0.101 | 0.316 | 0.921*** | 0.342 | 0.111 | -0.218 |
|  | (0.131) | (0.152) | (0.171) | (0.243) | (0.172) | (0.112) | (0.122) | (0.237) | (0.319) | (0.520) | (0.305) | (0.307) |
| Completed primary school | 0.064 | -0.124 | -0.346** | 0.126 | 0.095 | -0.192* | -0.158 | -0.050 | 0.422* | -0.780*** | 0.858* | -0.168 |
|  | (0.098) | (0.092) | (0.145) | (0.112) | (0.122) | (0.112) | (0.162) | (0.128) | (0.253) | (0.230) | (0.447) | (0.322) |
| Number of adults | 0.060*** | -0.048** | -0.054* | -0.066 | 0.058** | -0.103*** | -0.069* | -0.055 | 0.214*** | -0.076* | -0.357*** | 0.123* |
|  | (0.022) | (0.022) | (0.030) | (0.048) | (0.028) | (0.024) | (0.036) | (0.046) | (0.055) | (0.044) | (0.101) | (0.070) |
| Cultivated land | -0.042 | -0.020 | -0.025 | -0.199*** | -0.120 | -0.140 | -0.057 | -0.284*** | -0.208 | 0.326 | 0.014 | -0.143 |
|  | (0.055) | (0.096) | (0.111) | (0.073) | (0.149) | (0.120) | (0.115) | (0.097) | (0.220) | (0.211) | (0.217) | (0.276) |
| Square of total cultivated land | 0.003 | 0.017 | 0.013 | 0.016** | 0.011 | 0.032** | 0.011 | 0.018** | 0.004 | -0.009 | -0.011 | 0.018 |
|  | (0.005) | (0.011) | (0.009) | (0.007) | (0.015) | (0.014) | (0.010) | (0.008) | (0.016) | (0.022) | (0.017) | (0.025) |
| Months lived | 0.151*** | 0.131*** | 0.109*** | 0.140*** | 0.114** | 0.083*** | 0.090** | 0.172*** | -0.112* | 0.010 | -0.292*** | -0.039 |
|  | (0.017) | (0.022) | (0.029) | (0.039) | (0.051) | (0.029) | (0.044) | (0.035) | (0.065) | (0.051) | (0.077) | (0.043) |
| Livestock ownership | 0.019** | -0.006 | -0.004 | -0.007 | -0.009 | -0.014*** | -0.011 | -0.006 | 0.013 | -0.006 | 0.006 | 0.008 |
|  | (0.008) | (0.005) | (0.008) | (0.014) | (0.006) | (0.004) | (0.016) | (0.022) | (0.020) | (0.007) | (0.031) | (0.036) |
| Access to non-farm income | 0.285*** | 0.341*** | 0.618*** | 0.127 | 0.470*** | 0.374** | 0.321* | 0.040 | 0.951*** | 0.726*** | 0.027 | 0.150 |
|  | (0.070) | (0.126) | (0.198) | (0.114) | (0.082) | (0.174) | (0.168) | (0.148) | (0.177) | (0.245) | (0.420) | (0.231) |
| Agricultural implement index | -0.008 | 0.075** | 0.140*** | 0.019 | -0.027 | 0.069** | 0.076 | -0.058 | 0.024 | 0.340*** | 0.134 | 0.179** |
|  | (0.039) | (0.030) | (0.053) | (0.052) | (0.063) | (0.034) | (0.055) | (0.054) | (0.089) | (0.082) | (0.137) | (0.089) |
| Received credit | 0.178 | 0.364*** | 0.014 | -0.474* | 0.029 | 0.475*** | -0.046 | -0.287 | 0.138 | 0.470* | -0.265 | 0.319 |
|  | (0.136) | (0.129) | (0.095) | (0.245) | (0.140) | (0.152) | (0.111) | (0.258) | (0.384) | (0.251) | (0.239) | (0.383) |
| Mobile phone | 0.006** | 0.000 | 0.005 | -0.003 | 0.008** | -0.005 | -0.003 | 0.007 | 0.016 | -0.004 | -0.000 | -0.031 |
|  | (0.003) | (0.004) | (0.005) | (0.010) | (0.004) | (0.005) | (0.008) | (0.013) | (0.012) | (0.008) | (0.021) | (0.033) |
| Treated group | 0.162 | 0.112** | -0.086 | 0.214 | 0.058 | 0.205** | 0.203 | 0.216 | 0.456** | 0.709** | 0.526 | 0.530 |
|  | (0.183) | (0.045) | (0.215) | (0.152) | (0.160) | (0.080) | (0.470) | (0.180) | (0.210) | (0.299) | (1.075) | (0.404) |
| Applied organic fertilizer | -0.220*** | -0.028 | -0.008 | -0.034 | -0.198** | 0.047 | -0.033 | -0.118 | -0.046 | 0.287 | 0.692** | 0.164 |
|  | (0.079) | (0.168) | (0.083) | (0.130) | (0.079) | (0.186) | (0.154) | (0.155) | (0.266) | (0.304) | (0.299) | (0.254) |
| Practiced intercropping | -0.257 | -1.291*** | -0.507** | -0.520 | -0.396** | -1.205*** | -0.477 | 0.516 | 0.728 | -0.340 | -2.044** | 1.124 |
|  | (0.196) | (0.105) | (0.216) | (0.343) | (0.200) | (0.143) | (0.337) | (0.500) | (0.849) | (0.348) | (0.834) | (0.934) |
| Drought shock | -0.088 | -0.163 | -0.157 | -0.182 | -0.175 | -0.286 | -0.188 | -0.282* | -0.437 | -0.516* | -0.971* | -0.434 |
|  | (0.098) | (0.124) | (0.119) | (0.146) | (0.143) | (0.180) | (0.211) | (0.151) | (0.346) | (0.274) | (0.536) | (0.379) |
| Crop pests shock | 0.291*** | 0.190* | 0.391*** | -0.058 | 0.264** | 0.145 | 0.491*** | -0.032 | -0.305* | 0.358 | 1.321*** | -0.047 |
|  | (0.085) | (0.100) | (0.103) | (0.272) | (0.109) | (0.153) | (0.147) | (0.356) | (0.167) | (0.400) | (0.211) | (0.193) |
| Sold to main market | 0.012 | -0.014 | 0.009 | 0.017 | -0.005 | -0.006 | 0.047*** | 0.015 | -0.057 | -0.038 | 0.042 | 0.046 |
|  | (0.017) | (0.014) | (0.015) | (0.019) | (0.024) | (0.022) | (0.010) | (0.029) | (0.035) | (0.043) | (0.041) | (0.096) |
| Distance nearest asphalt road | 0.002 | 0.010 | -0.013 | 0.006 | 0.004 | 0.004 | -0.032** | 0.001 | 0.006 | 0.032 | -0.090*** | -0.011 |
|  | (0.007) | (0.007) | (0.008) | (0.012) | (0.008) | (0.007) | (0.013) | (0.012) | (0.016) | (0.020) | (0.030) | (0.019) |
| Access to markets | 0.000 | -0.001 | -0.003 | -0.002 | -0.001 | -0.001 | -0.004** | -0.000 | -0.003* | -0.005 | 0.004 | -0.002 |
|  | (0.001) | (0.001) | (0.002) | (0.003) | (0.001) | (0.002) | (0.002) | (0.003) | (0.002) | (0.004) | (0.004) | (0.005) |
| Manyara region | -0.115 | 0.198* | -0.240 | -0.301 | -0.317 | 0.004 | -0.587 | -0.409 | 1.141* | 1.546*** | 1.043 | 2.273*** |
|  | (0.173) | (0.104) | (0.338) | (0.375) | (0.255) | (0.142) | (0.395) | (0.373) | (0.646) | (0.238) | (1.311) | (0.555) |
| Constant | 11.156*** | 12.955*** | 13.064*** | 13.646*** | 10.949*** | 13.571*** | 13.325*** | 11.159*** | 3.353** | 6.580*** | 9.807*** | 7.510*** |
|  | (0.341) | (0.446) | (0.491) | (0.933) | (0.534) | (0.487) | (0.441) | (1.257) | (1.513) | (1.204) | (0.813) | (2.107) |

Standard errors corrected for intra-cluster correlation in parenthesis. * p<0.10, ** p<0.05, *** p<0.001. The base category (M_0_L_0_) is market non-participation; M_1_L_0_: only maize market participation; M_0_L_1_: only legume market participation; M_1_L_1_: joint maize and legume markets participation

A5 Table cont.: Determinants of income and food security (second stage results of the MIPWRA model)

| Variables | Months of food insecurity | | | | HFIAS | | | |
| --- | --- | --- | --- | --- | --- | --- | --- | --- |
|  | M_0_ L_0_ | M_1_ L_0_ | M_0_ L_1_ | M_1_ L_1_ | M_0_ L_0_ | M_1_ L_0_ | M_0_ L_1_ | M_1_ L_1_ |
| Sex of the household head | -1.340* | -0.174 | -0.204 | -0.445 | -0.628* | -0.140 | -1.313** | -0.108 |
|  | (0.714) | (0.369) | (1.104) | (0.437) | (0.359) | (0.192) | (0.544) | (0.178) |
| Completed primary school | -0.402 | 0.160 | -0.295 | 0.199 | -0.082 | 0.158 | -0.379 | 0.158* |
|  | (0.345) | (0.283) | (0.565) | (0.180) | (0.155) | (0.190) | (0.528) | (0.084) |
| Number of adults | -0.244*** | 0.160 | -0.000 | 0.045 | -0.092** | -0.063 | -0.097 | 0.224* |
|  | (0.062) | (0.152) | (0.140) | (0.062) | (0.036) | (0.070) | (0.078) | (0.116) |
| Total cultivated land (ha) | 0.459*** | 0.448 | -0.226 | -0.066 | 0.193* | -0.155 | -0.251 | -0.003 |
|  | (0.142) | (0.286) | (0.612) | (0.200) | (0.101) | (0.207) | (0.351) | (0.170) |
| Square of total cultivated land | -0.016 | -0.057* | 0.017 | 0.037 | -0.006 | 0.009 | 0.026 | 0.001 |
|  | (0.012) | (0.030) | (0.050) | (0.035) | (0.008) | (0.017) | (0.029) | (0.026) |
| Months lived | -0.101 | -0.018 | 0.027 | 0.079 | -0.043** | -0.144*** | -0.021 | 0.320** |
|  | (0.063) | (0.084) | (0.064) | (0.049) | (0.018) | (0.040) | (0.042) | (0.142) |
| Livestock ownership | -0.062*** | -0.007 | -0.072 | -0.008 | -0.040*** | -0.002 | 0.013 | 0.007 |
|  | (0.015) | (0.005) | (0.048) | (0.012) | (0.008) | (0.004) | (0.034) | (0.016) |
| Access to non-farm income | 0.435** | 0.011 | -0.562 | -0.159 | 0.036 | -0.410 | 0.531 | -0.124 |
|  | (0.216) | (0.404) | (0.373) | (0.144) | (0.095) | (0.260) | (0.332) | (0.154) |
| Agricultural implement index | -0.005 | -0.109 | -0.110 | -0.069 | 0.029 | -0.185*** | -0.250*** | 0.011 |
|  | (0.113) | (0.068) | (0.160) | (0.042) | (0.070) | (0.071) | (0.088) | (0.066) |
| Received credit | 0.031 | -0.459 | 1.044** | 0.179 | 0.271 | 0.332 | 0.314 | 0.151 |
|  | (0.326) | (0.324) | (0.421) | (0.154) | (0.215) | (0.293) | (0.200) | (0.296) |
| Mobile phone | -0.030** | 0.025 | 0.006 | 0.020 | -0.025*** | 0.009 | 0.048*** | -0.024 |
|  | (0.015) | (0.017) | (0.025) | (0.016) | (0.006) | (0.010) | (0.014) | (0.016) |
| Treated group | -0.026 | -0.351 | 0.501 | -0.124 | -0.109 | -0.374** | 0.236 | 0.241 |
|  | (0.536) | (0.615) | (1.074) | (0.134) | (0.176) | (0.151) | (0.549) | (0.180) |
| Applied organic fertilizer | 0.508 | -0.623 | -0.648 | -0.049 | 0.089 | -0.097 | -0.245 | -0.156 |
|  | (0.448) | (0.390) | (0.539) | (0.230) | (0.230) | (0.244) | (0.228) | (0.193) |
| Practiced intercropping | 0.873 | 0.748 | -0.393 | 0.552* | 0.124 | 0.340 | -0.338 | 0.469 |
|  | (0.705) | (0.472) | (0.639) | (0.330) | (0.456) | (0.224) | (0.213) | (0.377) |
| Drought shock | -0.357 | 0.657* | 1.965 | 0.406 | -0.015 | -0.260 | 0.220 | 0.283 |
|  | (0.224) | (0.393) | (1.616) | (0.338) | (0.211) | (0.170) | (0.704) | (0.189) |
| Crop pests shock | 0.457** | -0.075 | -1.588*** | 0.295** | 0.212 | 0.070 | -1.230** | 0.021 |
|  | (0.215) | (0.304) | (0.564) | (0.124) | (0.174) | (0.288) | (0.522) | (0.097) |
| Sold to main market | 0.061 | -0.067* | 0.025 | -0.003 | -0.015 | -0.002 | -0.049 | 0.001 |
|  | (0.058) | (0.039) | (0.077) | (0.032) | (0.021) | (0.032) | (0.034) | (0.034) |
| Distance nearest asphalt road | 0.005 | -0.006 | 0.080** | 0.010 | 0.010 | -0.009 | 0.058*** | -0.047 |
|  | (0.018) | (0.028) | (0.040) | (0.011) | (0.013) | (0.013) | (0.020) | (0.029) |
| Access to markets | -0.005*** | -0.002 | -0.005 | -0.004* | -0.002 | 0.000 | -0.010 | -0.003 |
|  | (0.002) | (0.004) | (0.014) | (0.002) | (0.001) | (0.002) | (0.009) | (0.003) |
| Manyara region | -1.317* | -0.560 | -1.989 | -0.332 | -0.571 | -0.588 | -3.037** | 0.080 |
|  | (0.683) | (0.570) | (1.595) | (0.379) | (0.368) | (0.435) | (1.221) | (0.402) |
| Constant | 5.338*** | -1.423 | 3.101 | -1.619 | 3.595*** | 0.861 | 1.917 | 0.570 |
|  | (1.237) | (1.744) | (3.237) | (1.343) | (0.708) | (0.921) | (1.332) | (1.320) |

Note: Standard errors corrected for intra-cluster correlation in parenthesis. * p<0.10, ** p<0.05, *** p<0.001. The base category (M_0_L_0_) is market non-participation; M_1_L_0_: only maize market participation; M_0_L_1_: only legume market participation; M_1_L_1_: joint maize and legume markets participation.


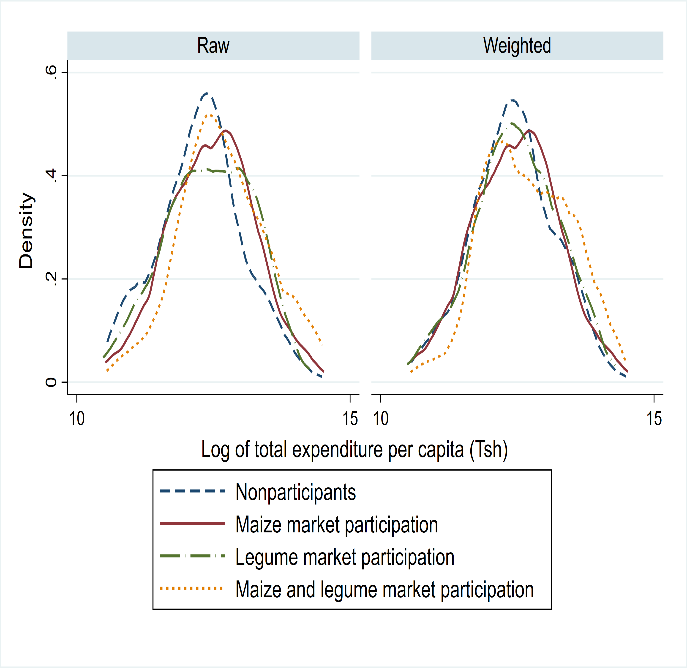

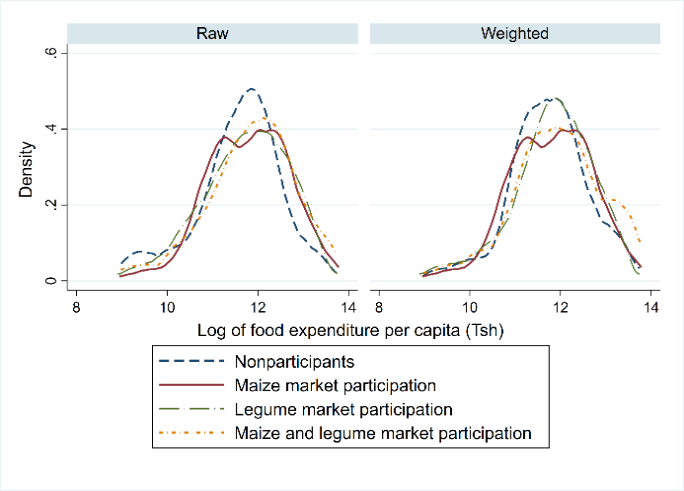

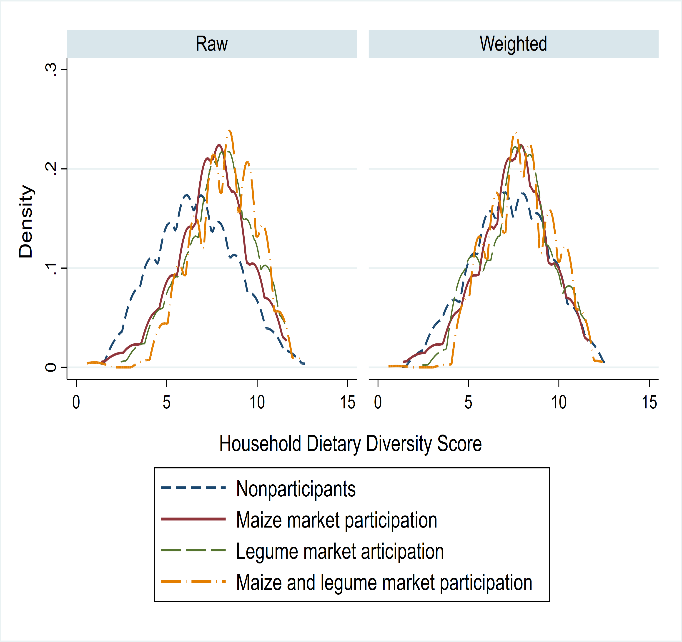

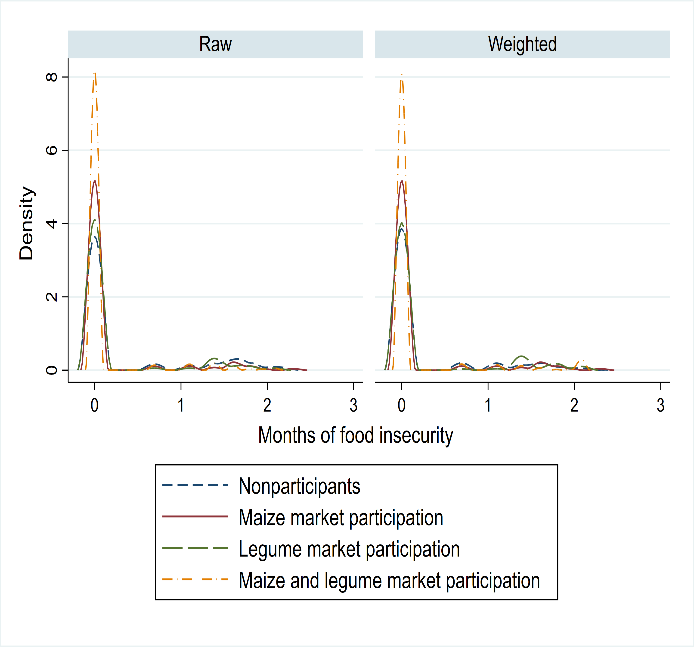

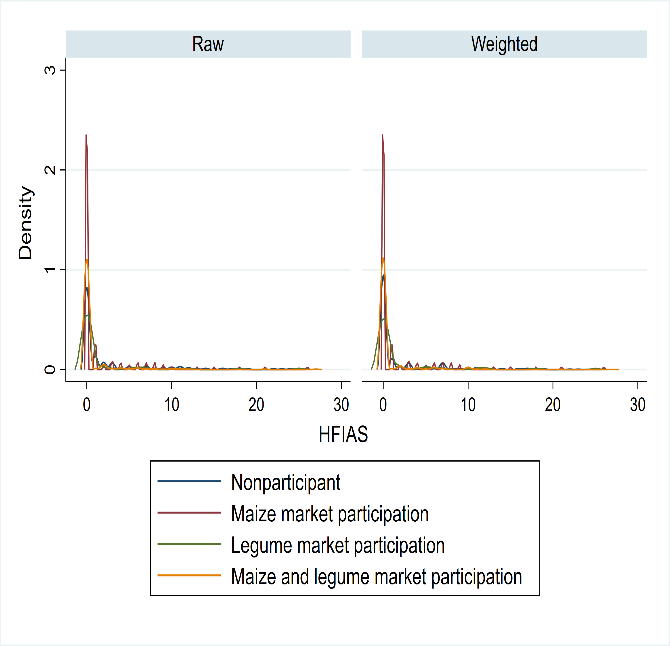


A1 Fig: Balanced plots for outcome variables by market participation
